# Inflammatory signatures for quick diagnosis of life-threatening infection during the CAR T-cell therapy

**DOI:** 10.1186/s40425-019-0767-x

**Published:** 2019-10-22

**Authors:** Hui Luo, Na Wang, Liang Huang, Xiaoxi Zhou, Jin Jin, Chunrei Li, Di Wang, Bin Xu, Jinhuan Xu, Lijun Jiang, Jue Wang, Yang Cao, Yi Xiao, Qian Zhang, Xia Mao, Songya Liu, Liting Chen, Min Xiao, Jianfeng Zhou

**Affiliations:** 1 Department of Hematology, Tongji Hospital, Tongji Medical College, Huazhong University of Science and Technology, Wuhan, 430030 Hubei China; 2 Immunotherapy Research Center for Hematologic Diseases of Hubei Province, Wuhan, 430030 Hubei China

**Keywords:** Chimeric antigen receptor-modified T-cell therapy, Infection, Cytokine release syndrome, Inflammatory factors

## Abstract

**Background:**

Chimeric antigen receptor-modified (CAR) T-cell immunotherapy is a novel promising therapy for treatment of B-cell malignancy. Cytokine release syndrome (CRS) and infection are the most common adverse events during CAR T-cell therapy. Similar clinical presentation of concurrent CRS and infection makes it difficult to differentially diagnose and timely treat the condition.

**Methods:**

We analyzed the features of infection events during the first 30 days after CAR T-cell infusion (CTI) in 109 patients from three clinical trials (ChiCTR-OPN-16008526, ChiCTR-OPC-16009113, ChiCTR-OPN-16009847). Based on the dynamic changes of interleukin (IL)-6 and ferritin, we proposed the “double peaks of IL-6” pattern as a feature of life-threatening infection during the first 30 days after CTI. Meanwhile, we screened candidate biomarkers from 70-biomarker panel to establish a prediction model for life-threatening infection.

**Results:**

In this study, 19 patients (17.4%) experienced a total of 19 infection events during the first 30 days after CAR T-cell infusion. Eleven patients (10.1%) had grade 4–5 infection, which were all bacterial infection and predominantly sepsis (*N* = 9). “Double peaks of IL-6” appeared in 9 out of 11 patients with life-threatening infection. The prediction model of three-cytokines (IL-8, IL-1β and interferon-γ) could predict life-threatening infection with high sensitivity (training: 100.0%; validation: 100.0%) and specificity (training: 97.6%; validation: 82.8%). On base of the aforementioned methods, we proposed a workflow for quick identification of life-threatening infection during CAR T-cell therapy.

**Conclusions:**

In this study, we worked out two diagnostic methods for life-threatening infection during CAR T-cell therapy by analyzing inflammatory signatures, which contributed to reducing risks of infection-induced death.

## Introduction

Chimeric antigen receptor-modified (CAR) T-cell immunotherapy represents a novel promising treatment and has achieved impressive anti-tumor responses in patients with refractory or relapsed (r/r) B-cell malignancies [1–6]. In August 2017, the U.S. Food and Drug Administration (FDA) granted the first approval to tisagenlecleucel (Kymriah; Novartis), a form of CD19-targeted CAR T-cell therapy [7]. Nonetheless, widespread clinical application of CAR T-cell therapy has been hampered by its severe or even fatal toxicity. Clinical trials with tisagenlecleucel demonstrated that 63–73% of patients experienced grade ≥ 3 adverse events related to tisagenlecleucel and the most common grade ≥ 3 adverse events included cytokine release syndrome (CRS) (22–46%), cytopenia lasting more than 28 days (24–32%), infections (20–24%) and febrile neutropenia (14–35%) [4, 5].

CRS is principally associated with the activation of CAR T-cells and lysis of the target tumor cells after CAR T-cell infusion (CTI) and is characterized by elevation of various serum inflammatory factors accompanied by high fever [8–10]. Clinically, since infection mimics CRS in terms of elevated inflammatory factors and fever, the diagnosis of infection becomes difficult in the presence of CRS [9]. However, the management of CRS and infection is different. CRS can be successfully ameliorated with interleukin (IL)-6 receptor inhibitor and corticosteroid, while infection needs prompt initiation of antibiotic therapy [8–10]. Thus, it is necessary to distinguish between infection and CRS in order to give proper treatment during CAR T-cell therapy.

Multiple high-risk factors, such as prior cytotoxic treatment, persistent pancytopenia, impaired host immunity, severe CRS, etc., contribute to frequent occurrence of infection during CAR T-cell therapy. Previous studies showed that 23–42% of patients on CAR T-cell therapy suffered from infection during the first month after CTI and 31% of the patients had infection between day 31 and day 180 [11, 12]. The infection was mainly (17–32%) of bacterial nature during the first month after CTI. Grade 4–5 infection, such as severe sepsis, is associated with high mortality if not treated promptly. Many current diagnostic techniques for bacterial infection, such as blood culture and medical imaging are limited since they are time-consuming and less sensitive [13]. Therefore, it is urgent to develop new approaches for quick identification of grade 4–5 bacterial infection during CAR T-cell therapy, especially differential diagnosis between infection and CRS. It has been found that, in severe sepsis, interferon (IFN)-γ is rarely elevated significantly although IL-6 is very high, which is quite different from the inflammatory signatures of CAR T-cell induced CRS [14–16]. This finding suggests that inflammatory signatures might help in the quick diagnosis of severe infection during CAR T-cell therapy.

In this study, we explored new diagnostic methods for life-threatening infection during CAR T-cell therapy by analyzing the differences of inflammatory signatures between CRS and infection, with an attempt to minimize the risk of infection and maximize the efficacy of CAR T-cell therapy.

## Materials and methods

### Patients

We reviewed 109 consecutive patients with r/r B-cell malignancies receiving CART-cell therapy at Tongji Hospital, Huazhong University of Science & Technology, Wuhan, China, from October 2017 to July 2018. The subjects were on three clinical trials, which were registered with Chinese Clinical Trial Registry (ChiCTR, number ChiCTR-OPN-16008526, ChiCTR-OPC-16009113, ChiCTR-OPN-16009847). The three clinical trials were: anti-CD19 CAR (CAR19) and anti-CD22 CAR (CAR22) T-cell “Cocktail” (CAR19/22) therapy for r/r B-cell malignancies [17], anti B-cell maturation antigen CAR T-cell (CAR-BCMA) therapy for r/r plasma cell malignancies [18, 19] and adoptive CAR19/22 therapy post-autologous hematopoietic stem cell transplantation (HSCT) for r/r B-cell lymphoma (HSCT+CAR19/22) [20], respectively. Our study was conducted upon approval by the Institutional Review Board and informed consent was obtained from each individual in strict accordance with the principles stipulated in Declaration of Helsinki.

### CAR-T cell production

The third-generation CAR-CD19, CAR-CD22 and CAR-BCMA were separately encoded by a lentiviral vector containing single-chain variable fragments from murine monoclonal antibodies against human CD19, CD22 and BCMA, a CD8a hinge, the CD28 transmembrane regions, 4-1BB and CD3ζ chain (Additional file 1: Figure S1A). Autologous T cells were cultured with anti-CD3&CD28 antibody-conjugated microbeads (Thermo Fisher Scientific, USA) and IL-2 (R&D systems, USA). Lentivirus-mediated CAR transduction was performed 24 h after the culture [21]. Transfection efficiency, apoptosis and tumoricidal activity were used for quality-control [22]. The CART-cells were cultured for 14 days and tested for viability, mycoplasma, endotoxin and sterility before cell infusion.

### Clinical protocol of CAR T-cell therapy

Patients before CAR19/22 therapy received lymphodepletion chemotherapy with FC regimen (fludarabine at 25 mg/m^2^ and cyclophosphamide at 300 mg/m^2^) for 3 days (day − 4 to day − 2). Afterwards, CAR19 and CAR22 T cells were separately infused in 2 divided doses. Patients on CAR-BCMA therapy received FC regimens for 3 days (day − 4 to day − 2), followed by infusion of CAR-BCMA T-cells in 2–3 divided doses. Patients on HSCT+CAR19/22 therapy received BEAM regimen (bis-chloroethyl nitrosourea, etoposide, Ara-C and melphalan) for 5 days (day − 6 to day − 2), followed by autologous hematopoietic stem cell infusion (day − 1). Afterwards, CAR19 and CAR22 T-cells were separately infused in 2 divided doses. The first day of CAR T-cell infusion was taken as Day 0. The clinical protocol is detailed in Additional file 1: Figure S1B.

### Supportive care and antimicrobial prophylaxis

Supportive care and antimicrobial prophylaxis in the three CAR-T therapy groups were administered as follows: antimicrobial prophylaxis was routinely used which included teicoplanin 0.4 g daily, linezolid 0.6 g once every 12 h, tienam 1.0 g every 8 h and voriconazole 0.2 g once every 12 h when leukopenia developed after lymphodepletion. Intravenous immunoglobin 0.4 g/kg was administered once the serum immunoglobin was < 20 g/L; granulocyte colony-stimulating factor was subcutaneously given at 300 μg/day when CRS was relieved, until neutrophil count returned to normal. When infection was suspected, attending physician would adjust antimicrobial protocols according to patients’ conditions and institutional guidelines. Besides, patients with CAR19/22 and CAR-BCMA therapy were treated in laminar flow hood and patients with HSCT+CAR19/22 were in transplantation cabin.

### CRS grading

CRS was graded on a 1–5 point scale proposed by Lee et al. [10], with severe CRS rated 3–5. The beginning of CRS, defined by the appearance of CRS symptoms such as fever ≥38.0 °C, was designated the first day. The ending of CRS, defined as disappearance of fever or other CRS symptoms, was taken as the last day. Tocilizumab and/or corticosteroids were used for controlling severe CRS.

### Grading and diagnosis of infection

Infection was assessed on a 5-point scale against the Common Terminology Criteria of Adverse Events (CTCAE, version 4.0.3) [12, 23], with deaths listed as 5, life-threatening events as 4, severe conditions necessitating intravenous antibiotics, as 3, moderate symptoms requiring oral treatment as 2 and mild symptoms entailing no treatment as 1.

Infection events during the first 30 days after CTI were diagnosed based on clinical symptoms, laboratory tests, radiographic and microbiologic findings. Bacterial infection was diagnosed when the bacterial culture and pathogenic microorganism DNA/RNA high-throughput genetic test (PMseq, The Beijing Genomics Institute, China) yielded positive results. PMseq is a method that can identify 6868 pathogens by high-throughput next-generation sequencing [24]. The method description of PMseq was written in Additional file 1. Fungal infection was diagnosed on basis of proven or probable invasive fungal disease (IFI) against the 2008 revised criteria [25]. Viral infection was diagnosed based on positive results of specific viral nucleic acid test and PMseq. The time of infection events was the day when the diagnostic tests were performed.

### Clinical data collection

Clinical data were extracted from the medical records, including age, gender, treatment history, duration of neutropenia, CRS, infection events, therapeutic responses, etc. Dynamic changes of serum IL-6 and ferritin were recorded by clinical monitoring. Neutropenia was diagnosed when an absolute neutrophil count < 0.5 × 10^9^/L.

### Establishment of prediction model for grade 4–5 infection

A total of 81 serum specimens were obtained from 109 patients, including 10 specimens of grade 4–5 infection, 10 specimens of grade 3–5 CRS and 61 specimens of grade 1–2 CRS. Specimens were collected as follows: Since the onset of CRS and infection varied among different patients, we collected specimens during the first 30 days after CTI and retrospectively selected the samples that were collected at the peak of IL-6. In addition, 14 serum specimens collected from healthy donors served as controls. Serum specimens were diluted 2 folds before detection. A 70-biomarker panel (Meso Scale Discovery, Germany, Cat. K1508K) mainly containing various inflammatory factors was employed in discovery and training phases according to instructions (70 biomarkers were listed in Additional file 1: Figure S4). Relative changes (versus healthy donors) were calculated and log transformation was done before analysis. The Mann-Whitney unpaired test was used for the inter-group comparison. A stepwise logistic regression was utilized for making prediction model. For the detection of IL-8, IFN-γ and IL-1β in the prediction model, a new panel containing the three cytokines was designed and used for validation. Hosmer-lemeshow test [26] and receiver operating characteristic (ROC) curve were used to evaluate the prediction model.

### Statistical analysis

Cluster 3.0 and Tree-view software packages were used for cluster analysis and heatmap drawing. All statistical tests were two-sided, with a *P* < 0.05 considered to be statistically significant. Statistical analysis was performed by using IBM SPSS Statistics software (version 19). Adobe Illustrator CS6 and GraphPad Prism 7 were employed for figure editing.

## Results

### Characteristics of patients

In this study, 109 adult patients from three clinical trials were pooled, including 84 patients on CAR19/22 therapy, 16 on CAR-BCMA therapy and 9 on HSCT+CAR19/22 therapy. The patients were diagnosed with relapsed (74.3%) or refractory (25.7%) B-cell malignancies before CART-cell treatment. The median age was 47 years (range: 15–67 years). 42 patients (38.5%) were female and 25 patients (23.0%) previously received autologous or allogeneic HSCT. The median infusion doses of CAR19, CAR22, CAR-BCMA were separately 4.0 × 10^6^, 4.6 × 10^6^, 9.9 × 10^6^ cells/kg. The median neutropenia duration lasted 11 days (range: 0–30 days) within the first 30 days after CTI. A total of 105 patients (96.3%) underwent CRS. 11 patients (10.4%) developed grade 3–5 CRS; one patient died of severe CRS. The median time of CRS beginning in three studies was all on day 2 after CTI and the median time of CRS ending was separately day 9, day 8, day 7 in CAR19/22, CAR-BCMA and CAR19/22+ HSCT (Additional file 1: Figure S5B). The occurrence time of CRS in 3 studies has no significant difference. Kinetics of CAR T-cells in each therapy group within the 30 days after CTI were shown in Additional file 1: Figure S6. Despite different designs and types of CAR-T cells used in three therapy groups, the CAR copies reached to a peak within 2 weeks after CTI, which was consistent with the occurrence time of CRS. Overall response rates of CAR19/22, CAR-BCMA, HSCT+CD19/22 were separately 83.3, 81.3, 88.9%, which were assessed in the first month after CTI. The patient data in each therapeutic group were presented in Table 1.

**Table 1:** Clinical Data of Patients on CAR T-Cell Therapy

	CAR19/22 (*N* = 84)	CAR-BCMA (*N* = 16)	HSCT + CAR-19/22 (*N* = 9)	Total (*N* = 109)
Age				
Years, median (range)	47 (15–67)	55 (34–69)	40 (25–61)	47 (15–67)
Sex				
Female	38 (45.2)	3 (18.8)	1 (11.1)	42 (38.5)
Diseases				
B-ALL	25 (29.8)	–	–	25 (22.9)
B-cell lymphoma	59 (70.2)	–	9 (100.0)	68 (62.4)
MM	–	16 (100.0)	–	16 (14.7)
Refractory or relapse				
Primary refractory	21 (25.0)	3 (18.8)	4 (44.4)	28 (25.7)
First relapse	30 (35.7)	3 (18.8)	1 (11.1)	34 (31.2)
≥ Second relapse	33 (39.3)	10 (62.5)	4 (44.4)	47 (43.1)
Prior HSCT				
Autologous	15 (17.9)	7 (43.8)	0 (0.0)	22 (20.2)
Allogeneic	3 (3.6)	0 (0.0)	0 (0.0)	3 (2.8)
CAR-T cell dose, ×10^6^ cells/kg, median (range)				
CART19	4.0 (1–10.0)	–	3.3 (1.8–10.0)	4.0 (1.0–10.0)
CART22	4.8 (1–13.5)	–	4.2 (1.8–10.0)	4.6 (1.0–13.5)
CART-BCMA	–	9.9 (5.4–20.0)	–	9.9 (5.4–20.0)
Neutropenia duration				
Days, median (range)	12 (0–30)	7 (2–16)	11 (2–15)	11 (0–30)
CRS grading				
Grade 0–2	75 (89.3)	14 (87.5)	9 (100.0)	98 (89.9)
Grade 3–5	9 (10.7)	2 (12.5)	0 (0.0)	11 (10.1)
Overall response rate^a^	70 (83.3)	13 (81.3)	8 (88.9)	91 (83.5)

Data are presented as number of patients (percentage in each therapeutic group) unless otherwise indicated. ^a^Overall response rate was assessed in the first month after CTI

### Infection events during the first 30 days after CTI

Overall, 19 patients (17.4%) experienced a total of 19 infection events during the first 30 days after CTI. Infection events in each therapeutic group are listed in Table 2. Predominant infection microorganisms were bacteria (*N* = 14); rare were virus (*N* = 3) and fungi (*N* = 2). The most common bacterial infection site was bloodstream (*N* = 12), followed by lung (*N* = 5), intestinal tract (*N* = 1), skin and soft tissue infection (*N* = 1). Grade 1–2 infection (*N* = 3) were rarely diagnosed clinically; grade 3 infection (*N* = 5) were diagnosed, including cytomegaloviremia (*N* = 1), probable IFI (*N* = 1) and bacterial infection (*N* = 3); grade 4–5 infection (*N* = 11) were diagnosed and all were severe bacterial infection, including sepsis shock (*N* = 9) and bacterial lung infection (*N* = 2). Five patients died of grade 4–5 infection.

**Table 2:** Infection Events during the First 30 Days after CART-cell Infusion

Types	CAR19/22 (*N* = 84)	CAR-BCMA (*N* = 16)	HSCT + CAR-19/22 (*N* = 9)	Total (*N* = 109)
Any infection^†^	14 (16.7)	3 (18.8)	2 (22.2)	19 (17.4)
Infection grading				
Grade 1–2	3 (3.6)	0 (0.0)	0 (0.0)	3 (2.8)
Grade 3	2 (2.4)	1 (6.3)	2 (22.2)	5 (4.6)
Grade 4–5	9 (10.7)	2 (12.5)	0 (0.0)	11 (10.1)
Infection microorganisms				
Bacteria	10 (11.9)	3 (18.8)	1 (11.1)	14 (12.8)
Fungi	1 (1.2)	0 (0.0)	1 (11.1)	2 (1.8)
Virus	3 (3.6)	0 (0.0)	0 (0.0)	3 (2.8)
Infection site				
Lung	1 (1.2)	2 (12.5)	2 (22.2)	5 (2.8)
Bloodstream	11 (13.1)	1 (6.3)	0 (0.0)	11 (10.1)
Others	2 (2.4)	0 (0.0)	0 (0.0)	2 (1.8)

Data are presented as number of patients (percentage in each therapeutic group)

Positive results of microbiological tests in 19 patients with infection events were listed in Additional file 1: Table S1. Bacterial culture yielded positive results in samples from peripheral blood (*N* = 7), sputum (*N* = 3), catheter blood (*N* = 1) and cutaneous purulent secretion (*N* = 1). Six patients had multiple drug-resistant bacterial infection. Two patients with negative results of bacterial culture were detected with high copies of bacteria genome by PMseq. Viral infection in one case was clinically identified by cytomegalovirus-DNA quantitative test and the other two cases were detected with low copies of cytomegalovirus and/or human alpha-herpesvirus 1 by PMseq. Two fungal infection were respectively diagnosed by fungi detection in stool routine and G/GM test.

**Fig. 1:**
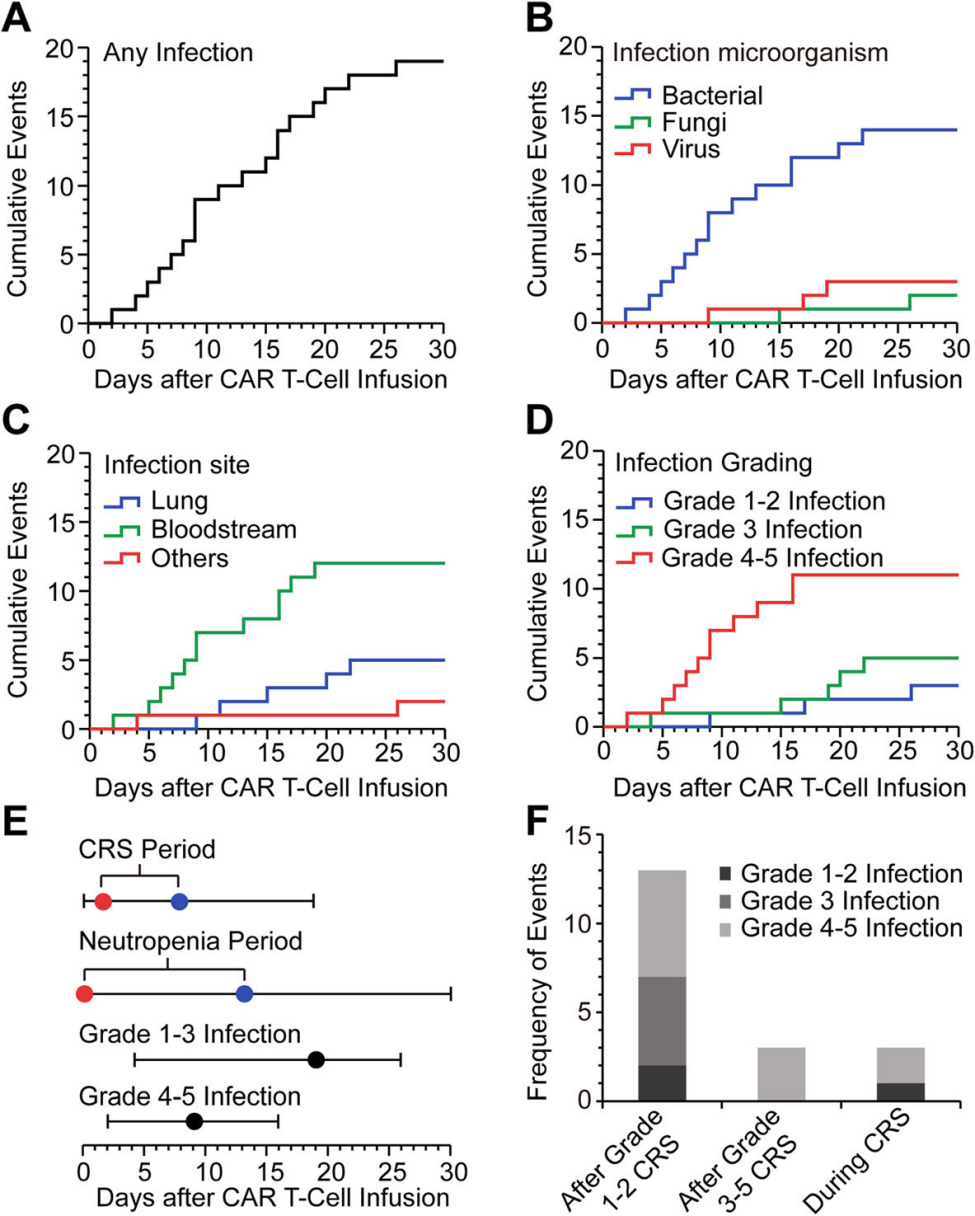
Infection events during the first 30 days after CTI. **a** The cumulative event curve of any infection events among all patients (*N* = 109). **b-d** The cumulative event curves of infection in terms of infectious microorganisms, infectious area or infection grades, respectively. **e** The occurrence time of CRS, neutropenia, grade 1–3 infection and grade 4–5 infection. Red dots (median, days) represent the beginning of CRS or neutropenia; blue dots (median, days) denote the ending of CRS or neutropenia; black dots (median, days) represent the occurrence time of grade 1–3 infection or grade 4–5 infection; lines show the ranges of events (days). **f** Frequency of infection events of various grades after grade 1–2 CRS or grade 3–5 CRS or during CRS

In this study, the time points of each infection event were shown by cumulative event curves (Fig. 1a-d). Viral infection and fungal infection were detected on day 17 (median, range: day 9 to day 26), which was later than the time of bacterial infection (median, day 9; range, day 2 to day 22) (Fig. 1b). The grade 4–5 infection took place mainly within the first 2 weeks after CTI (median, day 9; range, day 2 to day 16), which was earlier than that of grade 1–3 infection (median, day 18; range, day 4 to day 26) (Fig. 1d). Grade 4–5 infection occurred much close to the CRS period (the onset at a median of day 2; the ending at a median of day 8) and mostly in neutropenia period (the onset at a median of day 0; the ending at a median of day 13) (Fig. 1e). Although most of infection events appeared after CRS period, 5 infection events occurred during CRS period (Fig. 1f). Most of infection events appeared after grade 1–2 CRS (*N* = 13) and 3–5 CRS period (*N* = 3); while 3 infection events occurred during CRS period (Fig. 1f).

### “Double peaks of IL-6” as a specific sign for grade 4–5 infection

**Fig. 2:**
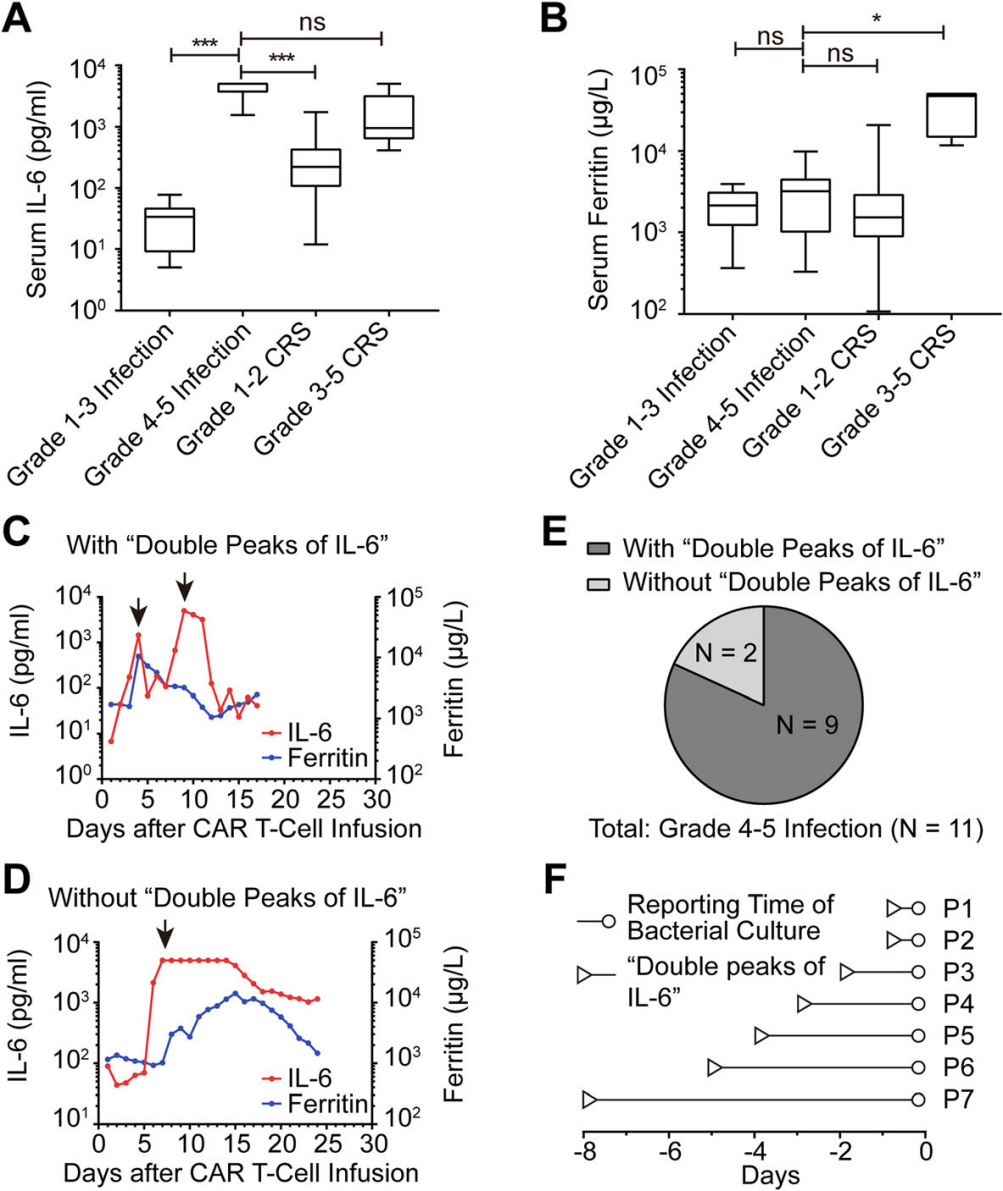
“Double Peaks of IL-6” as a sign for grade 4–5 infection **a-b** The peak level of serum IL-6 and ferritin during the period of grade 1–3 infection, grade 4–5 infection, grade 1–2 CRS and grade 3–5 CRS. Data were statistically analyzed by Mann-Whitney tests; ns, not significant; *, *P* < 0.05; ***, *P* < 0.001. **c-d** Dynamic changes of serum IL-6 and ferritin in two patients with grade 4–5 infection. The arrows represent the peaks of IL-6; In the “double peaks of IL-6”, the first peak appeared during CRS period and the second peak took place during the period of grade 4–5 infection. In the absence of the “double peaks of IL-6”, the only peak of IL-6 occurred during the period of concurrent grade 4–5 infection and CRS. **e** The frequency of “double peaks of IL-6” in patients with grade 4–5 infection (*N* = 11). **f** The occurrence time of “double peaks of IL-6” relative to the reporting time of positive bacterial culture in 7 patients with grade 4–5 bacterial infection

In all patients, serum IL-6 and ferritin were dynamically observed and we found that grade 4–5 infection caused changes that were characteristically different from CRS. To explore the differences, we compared the peak levels of serum IL-6 and ferritin among different groups (Fig. 2a-b). Although the grade 4–5 infection and grade 3–5 CRS groups had much higher serum IL-6 compared to grade 1–3 infection and grade 1–2 CRS groups, only grade 3–5 CRS had significant concurrent elevation of ferritin (Additional file 1: Figure S3), which was not observed in grade 4–5 infection group. Dynamic observation of serum IL-6 and ferritin revealed two patterns in patients with grade 4–5 infection. Typically, a CRS-associated elevation of serum IL-6 lasts for about a week after CTI and drops to baseline level, with CRS resolving by various clinical treatments. In most patients with grade 4–5 infection, a second IL-6 peak induced by grade 4–5 infection appeared immediately after the abrogation of the first CRS-related IL-6 peak. We designated the characteristic pattern of serum IL-6 as “double peaks of IL-6” (Fig. 2c and Additional file 1: Figure S2). The diagnostic criteria for “double peaks of IL-6” included: (1) serum IL-6 level in the first peak declined steadily due to relief of CRS; (2) IL-6 in the second peak increased abruptly to more than 1000 pg/ml; (3) the fluctuation of IL-6 level induced by pharmacologic management such as corticosteroid and/or tocilizumab was excluded; (4) ferritin increase was less than 50%. In this study, “double peaks of IL-6” pattern was found in 9 of 11 patients with grade 4–5 infection (Fig. 2e) and it was not observed in the other patients with CRS and/or grade 1–3 infection. Of the remaining 2 patients with grade 4–5 infection, only one IL-6 peak appeared when grade 4–5 infection and CRS occurred at the same time (Fig. 2d and Additional file 1: Figure S2). Compared to blood culture, diagnosis based on the “double peaks of IL-6” pattern was much quick and simple (a median of 3 days faster than blood culture in terms of reporting time) (Fig. 2f).

### Prediction model of three-cytokines for grade 4–5 infection

**Fig. 3:**
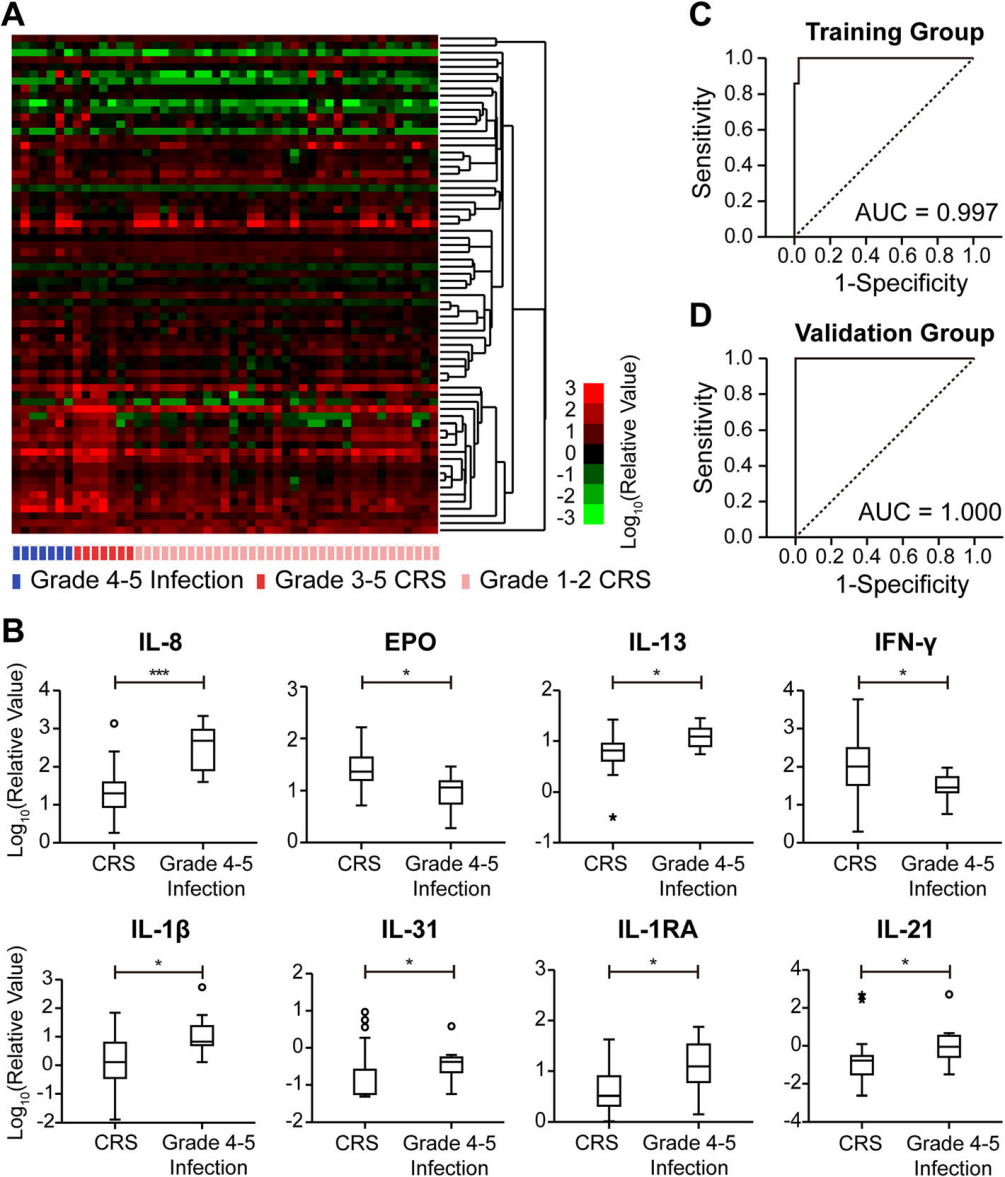
Prediction model for grade 4–5 infection. **a** Relative level of serum 70 biomarkers (versus healthy donors) was shown by a heatmap after unsupervised clustering analysis. **b** The biomarkers that showed statistical differences in serum levels between patients with CRS and those with grade 4–5 infection (IL-8, EPO, IL-13, IFN-γ, IL-1β, IL-31, IL-1RA, IL-21). The data were statistically analyzed by Mann-Whitney tests; *, *P* < 0.05; ***, *P* < 0.001. **c-d**. To assess the prediction model of three-cytokines (IL-8, IFN-γ and IL-1β), ROC analysis was performed in training group and validation group

Elevation of various inflammatory factors is a common clinical presentation of CRS and grade 4–5 infection. The severity of CRS was reportedly related to the level of some serum inflammatory factors, which were used in multiple prediction models for severe CRS [27, 28]. In Additional file 1: Figure S5C, the heatmap described the 20 pro-inflammatory and anti-inflammatory cytokine spectra of CRS from 3 studies, which revealed that the decreasing and increasing tendency of cytokines were uniform. Principal component analysis (PCA) revealed that cytokine spectra of CRS from 3 studies were not substantially different (Additional file 1: Figure S5D). Under this premise, we put three therapy groups together for difference analysis of CRS and infection. In the present study, we sought to establish a prediction model for grade 4–5 infection based on the hypothesis that grade 4–5 infection and CRS-induced inflammatory factor profiles might be different. Forty-nine serum specimens, including 7 specimens of grade 4–5 infection, 7 specimens of grade 3–5 CRS and 35 specimens of grade 1–2 CRS, were examined by a 70-biomarker panel during discovery and training phases. Meanwhile, the remaining 32 specimens, including 3 specimens of grade 4–5 infection, 3 specimens of grade 3–5 CRS and 26 specimens of grade 1–2 CRS, were used for validation. In discovery phase, the relative levels of 70 biomarkers were presented by a heatmap after an unsupervised cluster analysis (Fig. 3a and cluster tree is given in Additional file 1: Figure S4). For preliminary screening, we compared the relative levels of 70 biomarkers between grade 4–5 infection and CRS groups by employing Mann-Whitney test. There were significant differences in the serum levels of IL-8, EPO, IL-13, IL-1β, IL-31, IL-1RA, IL-21 and IFN-γ (*P* < 0.05: 0.000, 0.010, 0.014, 0.015, 0.020, 0.026, 0.034, 0.037, respectively) (Fig. 3b). Stepwise logistic regression was applied to develop a prediction model for grade 4–5 infection in the training phase based on significantly differential biomarkers aforementioned, and the following equation of the prediction model of three-cytokines was obtained: logit(*P*) = 6.394 + 19.035 × lg(relative_IL-8) + 13.789× lg(relative_IL-1β)-24.846 × lg(relative_IFN-γ), where, 6.394 is the intercept; lg (relative_IL-8), lg(relative_IL-1β) and lg(relative_IFN-γ) are the values of IL-8, IL-1β, IFN-γ after relative change (versus healthy donors) and log transformation; 19.035, 13.789 and − 24.846 are the slopes (coefficients), respectively, for lg(relative_IL-8), lg(relative_IL-1β) and lg(relative_IFN-γ). The cut-off value of logit (P) was 1.24 that maximized the sensitivity and specificity (Youden’s index [29]) to differentiate grade 4–5 infection and CRS. When logit (P) was greater than or equal to 1.24, the diagnosis of grade 4–5 infection could be made. The Hosmer-lemeshow test was applied to evaluate the fit of the model (chi-square = 0.183 with 8 df, *P* = 1.000). For ROC curve analysis, the area under the receiver operating characteristic curve (AUC) in training group was 0.997 (95% confidence interval [CI], 0.986 to 1.000; *P* < 0.001) (Fig. 3c). With the cut-off value, the prediction model had a sensitivity of 100.0% and a specificity of 97.6% in training group. The parameters of the prediction model from the training group were used to an independent cohort for validation. The AUC in validation group was 1.000 (95% CI, 1.000 to 1.000; *P* = 0.008) (Fig. 3d) with a sensitivity of 100.0% and a specificity of 82.8%. On the basis of the results, we were led to conclude that the prediction model of three inflammatory factors (IL-8, IL-1β and IFN-γ) had an excellent sensitivity and specificity for the prediction of grade 4–5 infection during the first 30 days after CTI.

### A workflow for quick diagnosis of grade 4–5 infection during the first 30 days after CTI

**Fig. 4:**
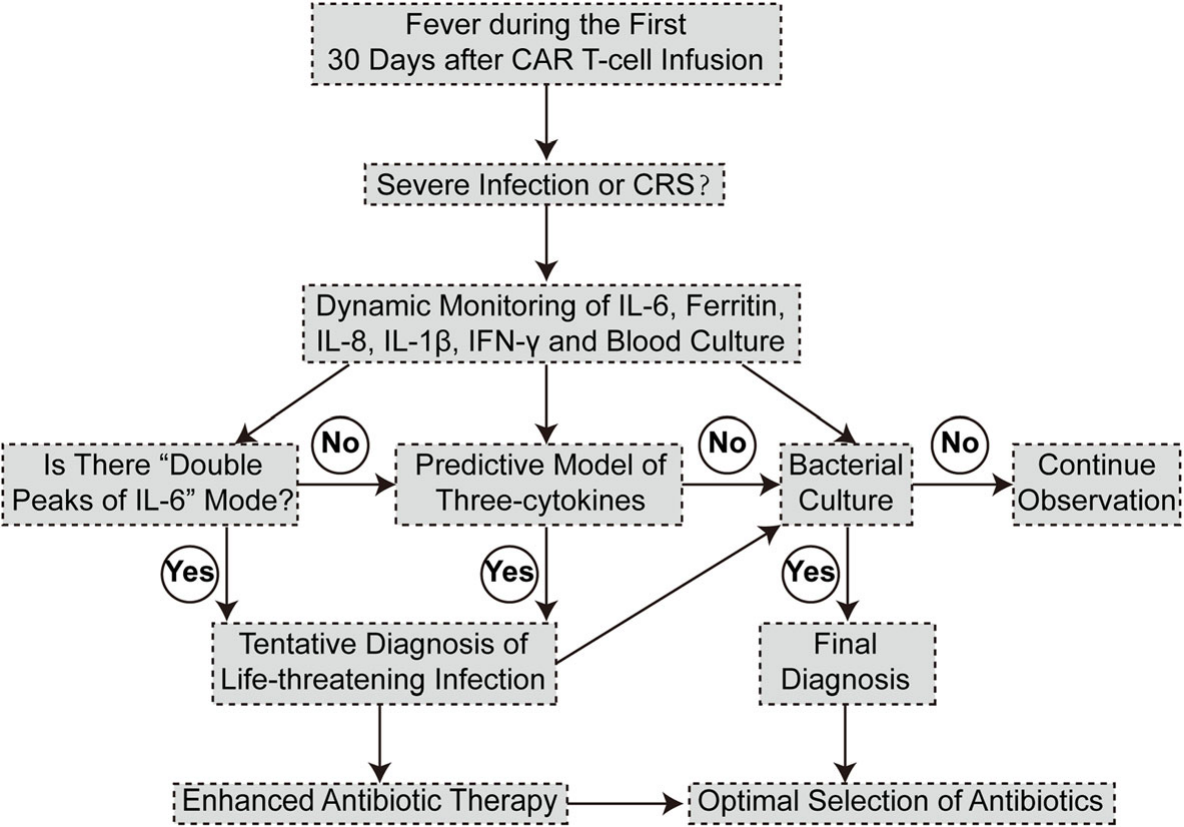
A workflow for quick identification of grade 4–5 infection during the first 30 days after CTI. Whenever patients had fever during the first 30 days after CTI, IL-6, ferritin, IL-8, IL-1β, IFN-γ and blood culture needed to be dynamically monitored during CAR T-cell therapy to distinguish CRS and severe infection. By means of the “double peaks of IL-6” plus the prediction model, we could tentatively diagnose grade 4–5 infection and immediately initiate enhanced antibiotic therapy. Bacterial culture would establish final diagnosis of bacterial infection

Based on the above-mentioned data, we proposed a workflow for early identification and intervention of grade 4–5 infection during the first 30 days after CTI (Fig. 4). Whenever patients have fever during the first 30 days after CTI, routine evaluation should be performed to identify the possibility of CRS and infection. In clinical practice, dynamic monitoring of IL-6, ferritin, IL-8, IL-1β, IFN-γ and blood culture is essential for the differential diagnosis. The appearance of “double peaks of IL-6” is an indicator of grade 4–5 infection. Meanwhile, the prediction model of three-cytokines (IL-8, IL-1β and IFN-γ) further confirms the diagnosis of grade 4–5 infection. Timely initiation of enhanced antibiotic therapy is imperative for these patients even in the absence of positive result of blood culture. Although detection reports take time, positive bacterial culture can help establish final diagnosis of bacterial infection and choose right antibiotics. If the three diagnostic means all yield negative results, patients should be monitored continuously and managed against the guidelines for CRS.

## Discussion and conclusions

In this study, we retrospectively analyzed the frequency, time distribution, types and severity of infection events during CAR T-cell therapy in our center. Our study showed that grade 1–3 infections were rarely detected and could be controlled under condition of prophylaxis with broad-spectrum coverage of antimicrobial agents during CAR T-cell therapy. The “collapse” of routine ant-infection defense led to the development of grade 4–5 infection. In this study, viral or fungal infections caused only mild or moderate infection with cytokines experiencing minor changes during the 30 days after CAR T-cell infusion. In contrast, all grade 4–5 infections were caused by bacterial infection, which resulted in a significant increase in inflammatory factors. Therefore, all grade 4–5 infections in this study could be taken as severe bacterial infection and caused the same change pattern of cytokines.

Previously, no studies differentially diagnosed CRS and infection by using inflammatory signatures, although CRS and infection severity could be separately predicted by inflammatory factors. In this study, we identified the “double peaks of IL-6” as a specific sign for grade 4–5 infection and developed the prediction model of three-cytokines (IL-8, IL-1β and IFN-γ) for diagnosing the grade 4–5 infection during the first 30 days after CTI. The appearance of “double peaks of IL-6” represented a “collapse” of routine ant-infection defense and an indicator of life-threatening infection with which an aggressive antibiotic therapy would be urgently needed even in the absence of positive blood culture. The three-cytokines model could separate CRS from severe infection when inflammatory factors were elevated. In some of cases, either CRS or infection are easy to manage. However, in some other cases, cytokine storms caused by strikingly elevated inflammatory factors are life-threatening and presented with fulminant clinical course, accompanied frequently by poor general conditions, multiple-organ failure as well as coagulopathy. An accurate diagnosis is urgently needed for a prompt CRS management or enhanced anti-microbial therapy. In this case, a practical method to differentiate CRS and infection can help physicians make a preliminary judgment within a very short therapeutic window. Importantly, the prediction model could also identify the grade 4–5 infection that might have been missed by “Double-peaks” diagnosis. Thus, double-peaks plus the prediction model, with the features of grade 4–5 infection taken into consideration, is of relevance for clinicians to quickly diagnosing life-threatening infection. Moreover, the workflow we proposed based on the two models can serve as a guide for clinicians.

Unlike other small-molecule or antibody therapies, CAR T-cell therapy is living-cell-based therapeutics, and CAR T-cells can rapidly proliferate and persist for a long time in vivo. This feature results in pharmacokinetics and toxicities different from those produced by traditional therapeutics. To better use CAR T-cell products, previous researches employed mathematical models to characterize CAR T-cell drugs, such as population pharmacokinetics (PPK) and population pharmacodynamics (PPD), prediction model of severe CRS [11, 12]. Our study suggested that distinguishing between grade 4–5 infection and CRS by mathematical prediction is feasible. In this study, we found that significantly higher level of serum IL-8 and IL-1β and lower level of serum IFN-γ were the hallmarks of grade 4–5 infection, and such profile of inflammatory factors was consistent with that of sepsis reported previously [14–16]. Such consistencies indicated that the mathematical prediction model involving IL-8, IFN-γ and IL-1β can well characterize the infection during CAR T-cell therapy. However, the nature of CART-cell products used by different settings varies substantially due to the differences in the cyto-dynamics of CART-cell products, diseases status and demographics of subjects, among others. Therefore, we can borrow a lot from their mathematical models, but we can’t fully copy them. The prediction model for diagnosing grade 4–5 infection was based on the data from patients treated in our center, with our home-made CAR cell-T products. The model can be extrapolated to other populations but should be tailored according to their specific populations.

In summary, this study provided two new approaches to identify grade 4–5 infection during the CAR T-cell therapy, which contributed to reducing risks of infection-induced death. This method has been used in our ongoing clinical trials (ChiCTR1900024088, ChiCTR1900023922, ChiCTR1800018137), aimed at the further verification of the clinical utility of these diagnostic tools. In the future, more investigations are warranted on the characteristics of CAR T-cell products to further improve their safety and effectivity.

## Additional file

**Additional file 1: Figure S1.** Schematic diagram of CARs structure and clinical protocol. **Figure S2.** Dynamic changes of serum IL-6 and ferritin in patients with grade 4–5 infection. **Figure S3.** Dynamic changes of serum IL-6 and ferritin in patients with grade 3–5 CRS. **Figure S4.** Cluster tree of heatmap in Fig. 3a. **Figure S5.** Characteristics and cytokine spectra of CRS in three CAR T-cell therapy groups. **Figure S6.** Kinetics of CAR T-cell in three CAR T-cell therapy groups. **Table S1.** Positive results of microbiological tests. Materials and methods: PMseq.

## Data Availability

Not applicable.
