# Supplementary material for: Inflammatory signatures for quick diagnosis of life-threatening infection during the CAR T-cell therapy
Source: J Immunother Cancer. 2019 Oct 22;7:271. doi: 10.1186/s40425-019-0767-x (PMC6806557; doi:10.1186/s40425-019-0767-x)
Supplement: Supplementary file 1 — Additional file 1: Figure S1. Schematic diagram of CARs structure and clinical protocol. Figure S2. Dynamic changes of serum IL-6 and ferritin in patients with grade 4–5 infection. Figure S3. Dynamic changes of serum IL-6 and ferritin in patients with grade 3–5 CRS. Figure S4. Cluster tree of heatmap in Fig. 3a. Figure S5. Characteristics and cytokine spectra of CRS in three CAR T-cell therapy groups. Figure S6. Kinetics of CAR T-cell in three CAR T-cell therapy groups. Table S1. Positive results of microbiological tests. Materials and methods: PMseq. [file 40425_2019_767_MOESM1_ESM.docx]

**Supplemental Materials**

CONTENTS:

Figure S1. Schematic diagram of CARs structure and clinical protocol……………………….2

Figure S2. Dynamic changes of serum IL-6 and ferritin in patients with grade 4-5 infection…4

Figure S3. Dynamic changes of serum IL-6 and ferritin in patients with grade 3-5 CRS……….5

FigureS4. Cluster tree of heatmap in Figure 3A……………………........……………….……6

Figure S5. Characteristics and cytokine spectra of CRS in three CAR T-cell therapy groups….7

Figure S6. Kinetics of CAR T-cell in three CAR T-cell therapy groups…………………….…8

Table S1. Positive results of microbiological tests………………………….….………………9

PMseq: pathogenic microorganism DNA/RNA high-throughput genetic test……………….10


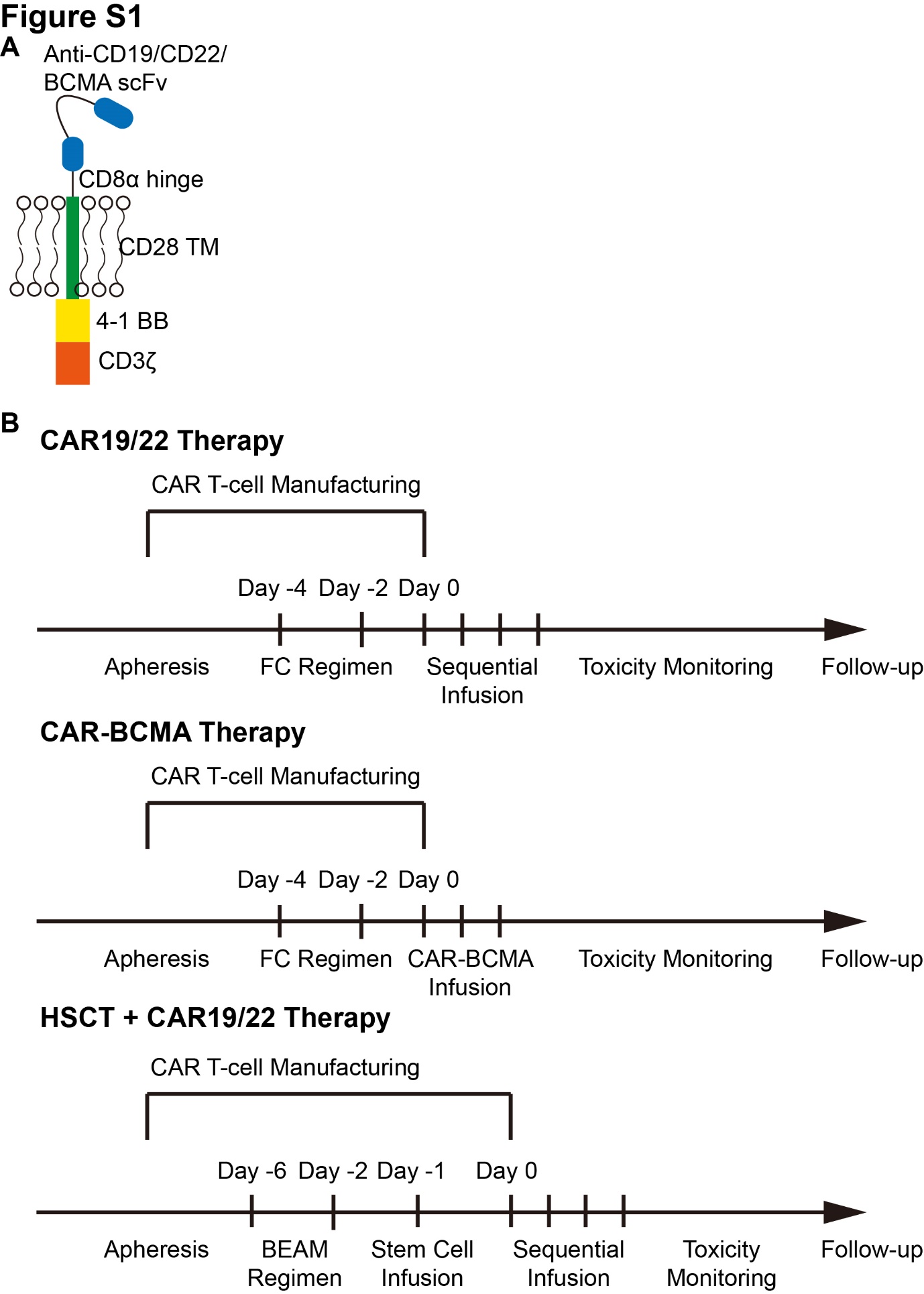


**Figure S1.** **Schematic diagram of CARs structure and clinical protocol.** (A) CAR consists of scFv (anti-CD19, anti-CD22 and anti-BCMA, respectively), CD8a hinge, CD28 transmembrane domain and 4-1BB/CD3ζ signaling domains. (B) Patients on CAR19/22 therapy received FC regimen for 3 days (day -4 to day -2). Afterwards, CAR19 and CAR22 T cells were separately infused in 2 divided doses. Patients on CAR-BCMA therapy received FC regimen for 3 days (day -4 to day -2). Afterwards, CAR-BCMA T cells were infused in 2-3 divided doses. Patients on HSCT+CAR19/22 therapy received BEAM regimen for five days (day -6 to day -2) followed by hematopoietic stem cells infusion (day -1). Afterwards, CAR19 and CAR22 T-cells were separately infused in 2 divided doses.


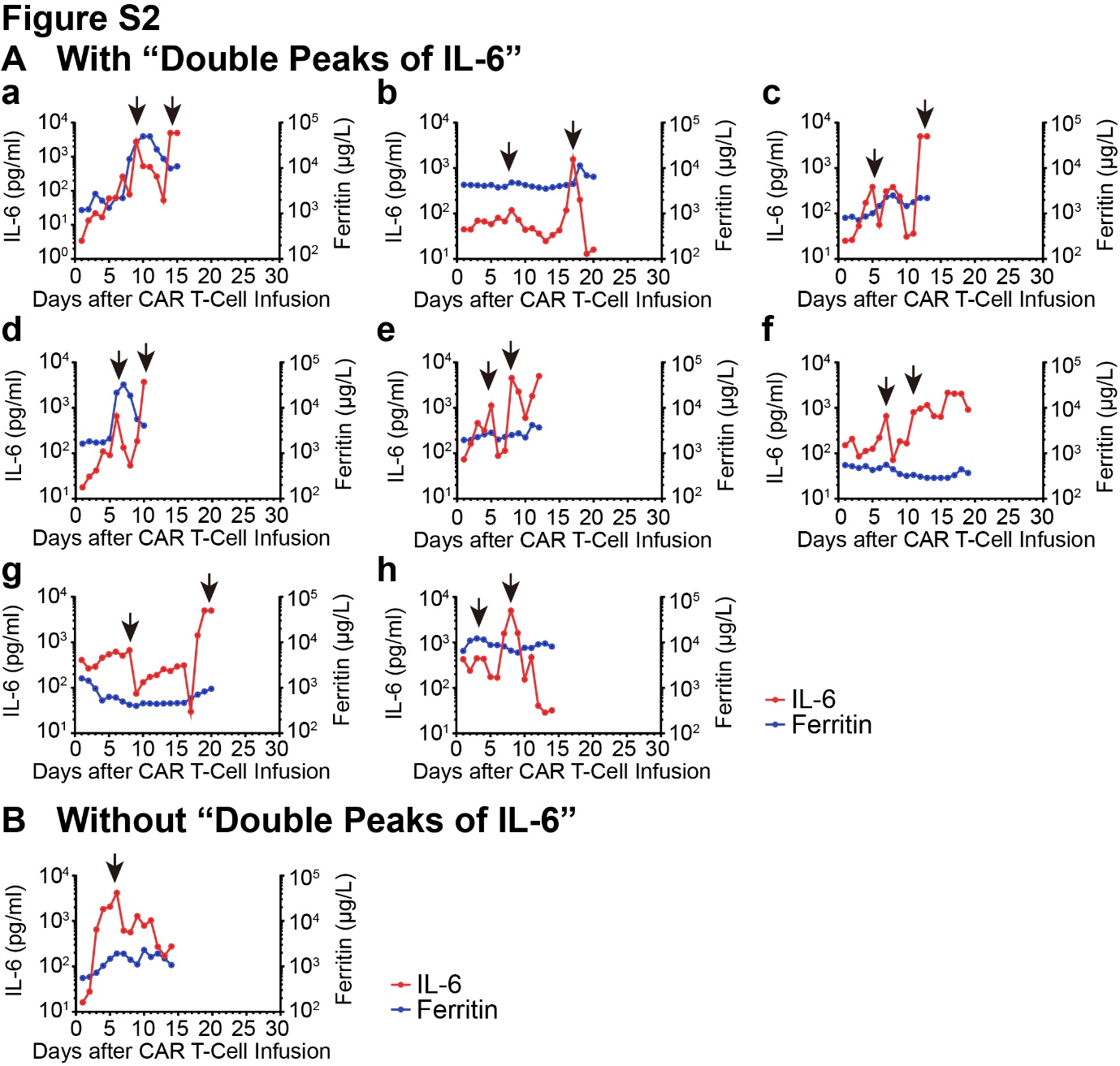


**Figure S2. Dynamic changes of serum IL-6 and ferritin in patients with grade 4-5 infection.** (A) eight patients with grade 4-5 infection during 30 days after CTI had the pattern of “Double peaks of IL-6”. (B) one patient with grade 4-5 infection didn’t have “Double peaks of IL-6”. The arrows represent the peaks of IL-6; in “Double peaks of IL-6” pattern, the first peaks were during CRS period and the second peaks were during the period of grade 4-5 infection; In Non- “Double peaks of IL-6” pattern, the peak of IL-6 was during the period of concurrent grade 4-5 infection and CRS.


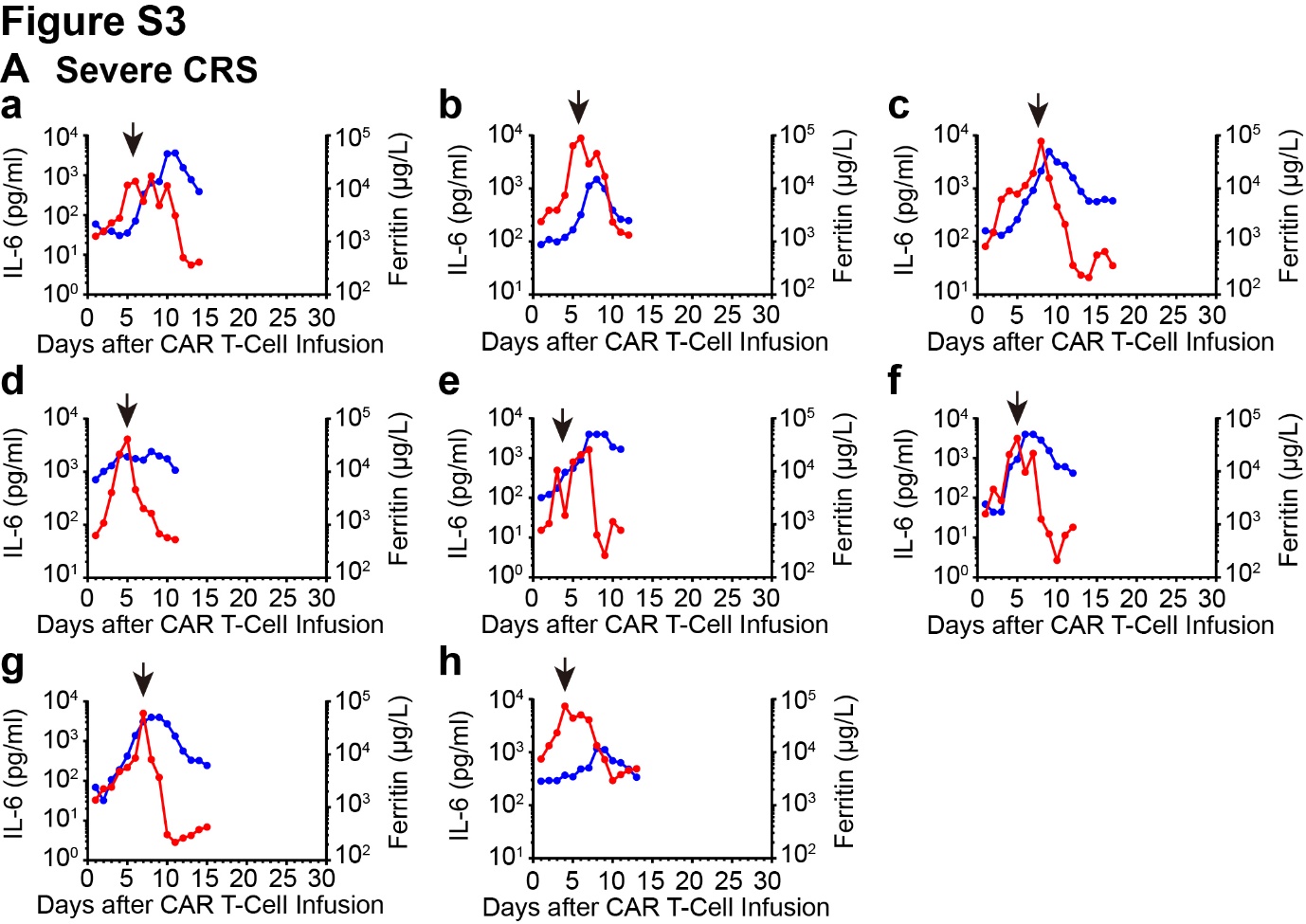


**Figure S3. Dynamic changes of serum IL-6 and ferritin in patients with grade 3-5 CRS.** The arrows represent the peaks of IL-6 during the period of grade 3-5 CRS without grade 4-5 infection.


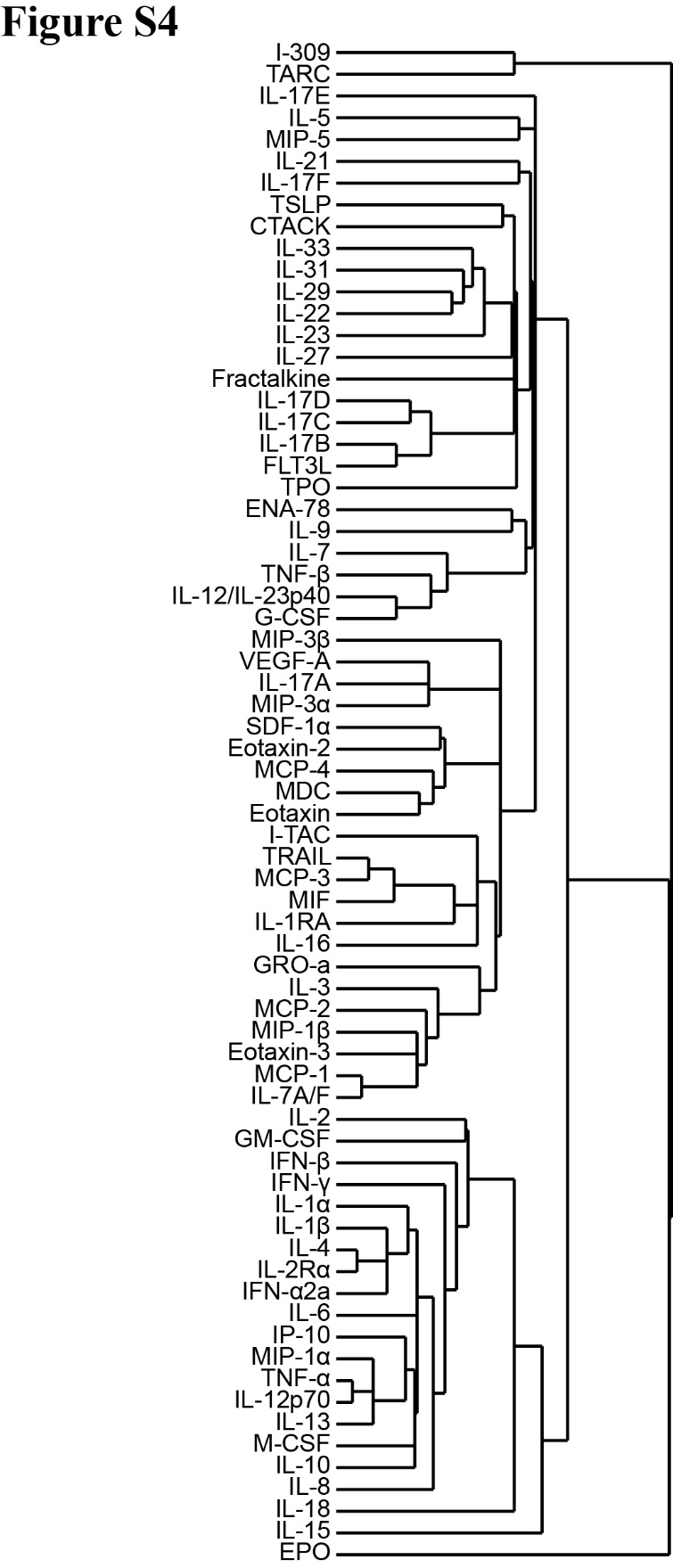


**Figure S4. Cluster tree of heatmap in Figure 3A.** Biomarkers in the cluster tree of Figure 3A were detailly listed.


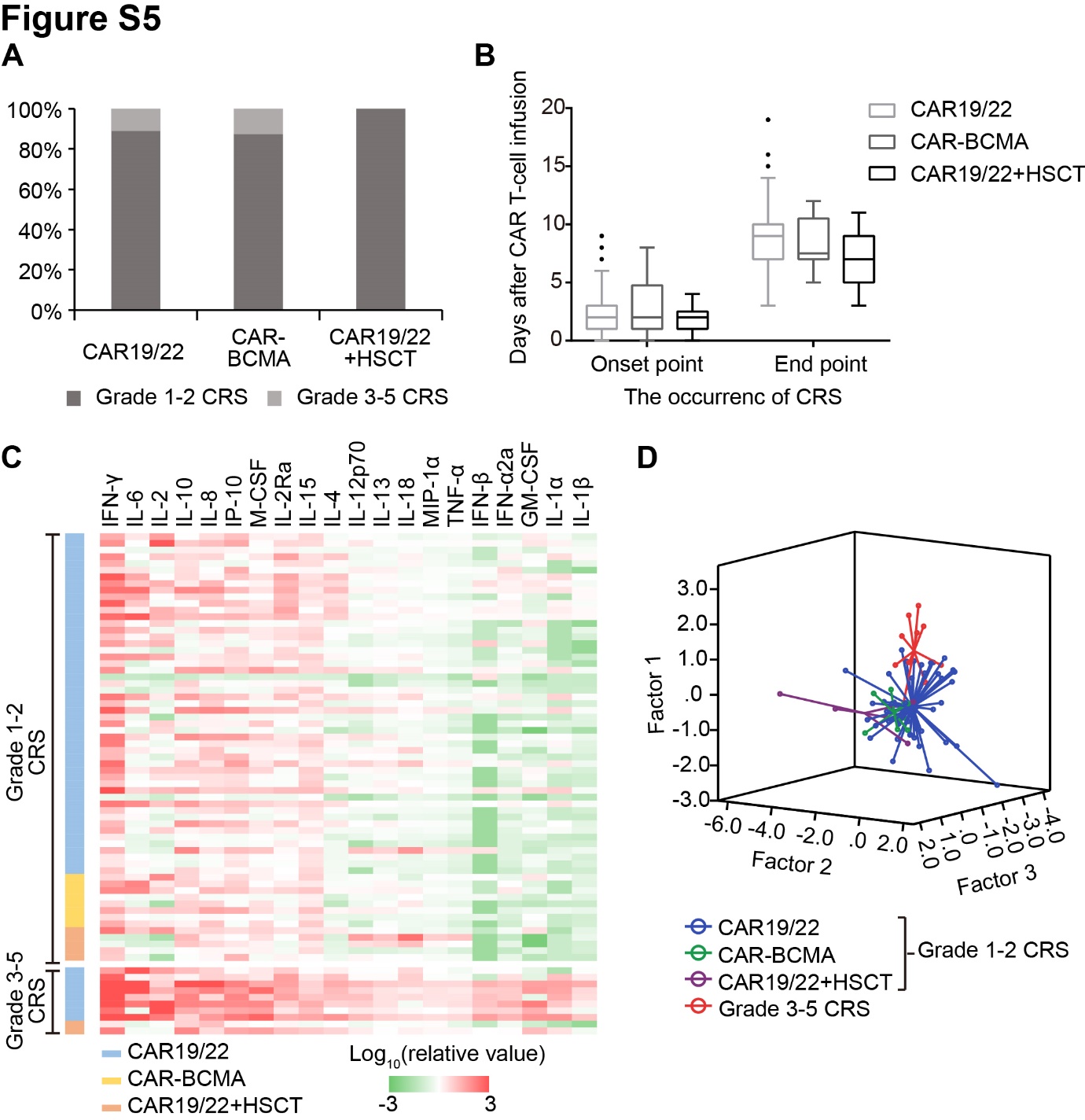


**Figure S5. Characteristics and cytokine spectra of CRS in three CAR T-cell therapy groups.** (A) Occurrence proportion of grade 1-2 and grade 3-5 CRS in patients with CAR19/22, CAR-BCMA and CAR19/22+HSCT therapy. (B) The onset and end point of CRS after CAR T-cell infusion in CAR19/22, CAR-BCMA and CAR19/22+HSCT. (C) Cytokine spectra in serum of patients with CAR19/22, CAR-BCMA and CAR19/22+HSCT therapy. (D) Three-dimensional scatter diagram built by three factors extracted from cytokine spectra by principal component analysis.


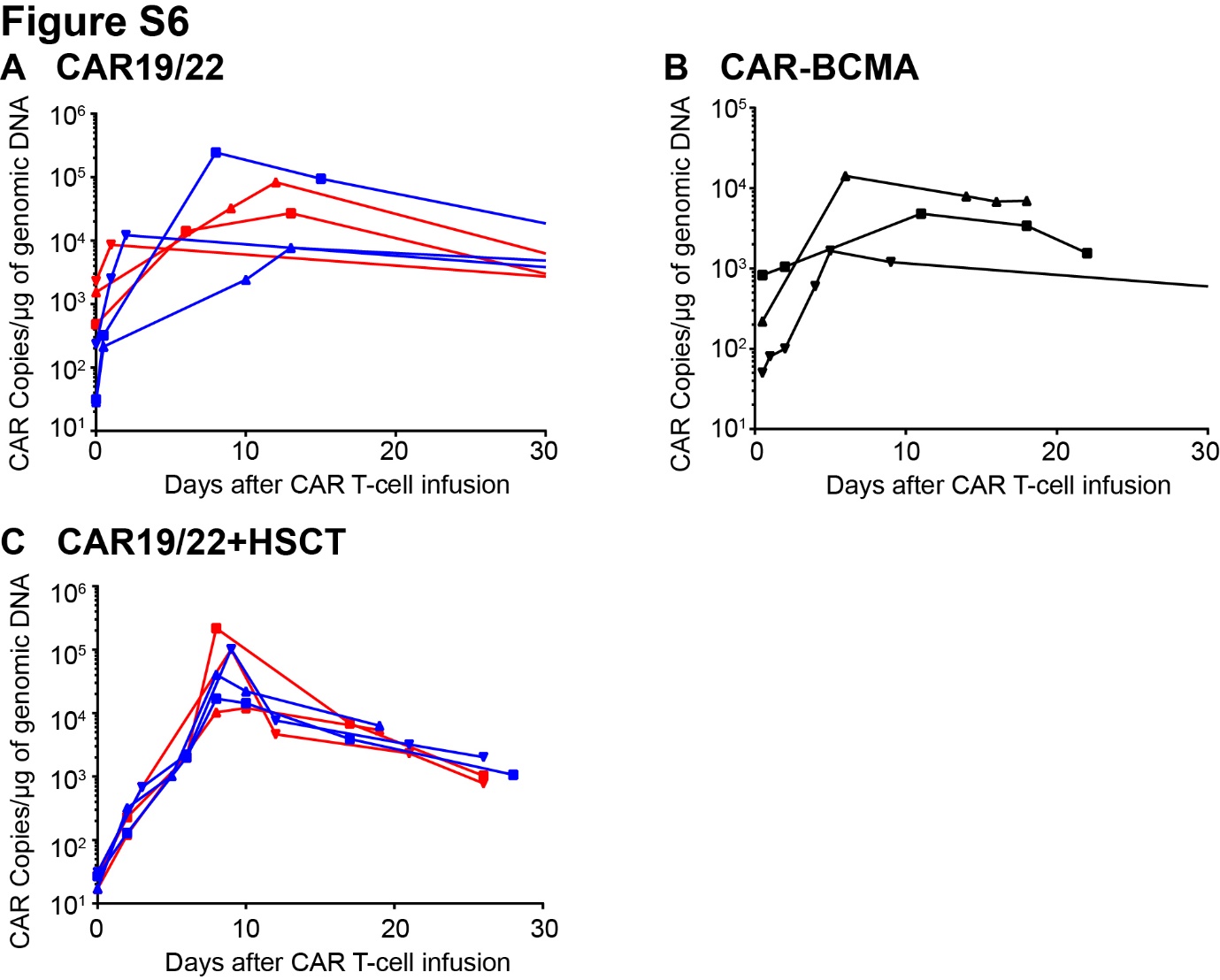


**Figure S6.** **Kinetics of CAR T-cell in Three CAR T-cell therapy groups.** (A) Kinetics of CAR19 and CAR22 in peripheral blood of 3 patients with CAR19/22 therapy. (B) Kinetics of CAR-BCMA in peripheral blood of 3 patients with CAR-BCMA therapy. (C) Kinetics of CAR19 and CAR22 in peripheral blood of 3 patients with CAR19/22+HSCT therapy. Curves of three shapes represent 3 patients. Red curves represent CAR19 and blue curves represent CAR22 in Figure S6A and Figure S6C. The CAR copies were detected by ddPCR.

| **Table S1. Positive results of microbiological tests** | | |
| --- | --- | --- |
| **Patients** | **Microbiological Tests** | **Results** |
| CART2 | Stool Routine | Fungi |
| CART3 | G/GM test | Positive |
| CART11 | PMseq | Lactobacillus iners (16514) Human alphaherpesvirus 1 (140) |
| CART16 | Blood culture | Stenotrophomonas maltophilia |
| CART23 | Sputum culture | Pseudomonas aeruginosa |
| CART26 | PMseq | Cytomegalovirus (76)  Human alphaherpesvirus 1 (31) |
| CART46 | Blood culture | Stenotrophomonas maltophilia |
| CART50 | Blood culture | Acinetobacter baumannii |
| CART52 | Sputum culture | Pseudomonas aeruginosa |
| CART53 | Blood culture | Burkholderia |
| CART55 | Culture of Skin purulent secretion | Pseudomonas aeruginosa |
| CART62 | PMseq  Cytomegalovirus-DNA quantitative test | Cytomegalovirus (233)  Human alphaherpesvirus 1 (50) 2.5×10^3^ Copies/ml |
| CART71 | PMseq | Cytomegalovirus (11) |
| CART75 | Blood culture | Klebsiella pneumoniae |
| CART79 | PMseq | Stenotrophomonas maltophilia (2940) |
| CART84 | Blood culture | Klebsiella pneumoniae |
| CART94 | Blood culture of PICC | Staphylococcus saprophyticus |
| CART98 | Sputum smear | Gram-negative bacteria |
| CART101 | Blood culture | Micrococcus luteus |
| G/GM test: 3-β-D Glucan test and galactomannan antigen test; PMseq: pathogenic microorganism DNA/RNA high-throughput genetic test; PICC: peripherally inserted central catheter. Numbers in brackets: the total number of microbial nucleic acid sequences detected by PMseq in serum. | | |

**PMseq: pathogenic microorganism DNA/RNA high-throughput genetic test**

DNA was extracted from 300 uL of serum using the TIANGEN Micro DNA Kit (DP316, TIANGEN BIOTECH, China) following the manufacturer’s instruction. RNA was extracted from 300 ul of serum using the TIANGEN RNA simple Kit (DP419, TIANGEN BIOTECH, China) DNA libraries and reversely transcribed into cDNA. DNA and cDNA libraries were constructed through DNA-fragmentation, end-repair, adapter-ligation and PCR amplification. Quality qualified libraries were sequenced by BGISEQ-50 platform. High-quality sequencing data were classified by simultaneously aligning to four Microbial Genome Databases, consisting of viruses, bacteria, fungi, and parasites. The classification reference databases were downloaded from NCBI (<ftp://ftp.ncbi.nlm.nih.gov/genomes/>). RefSeq contains 4,061 whole genome sequence of viral taxa, 2,473 bacterial genomes or scaffolds, 199 fungi related to human infection, and 135 parasites associated with human diseases.
